# The influence of distracter and target features on distracter induced blindness

**DOI:** 10.2478/v10053-008-0103-3

**Published:** 2012-02-15

**Authors:** Lars Michael, Markus Kiefer, Michael Niedeggen

**Affiliations:** 1 Department of Educational Science and Psychology, Freie Universität Berlin, Germany; 2 Department of Psychiatry, University of Ulm, Germany

**Keywords:** distracter induced blindness, task set inhibition, selective attention

## Abstract

The inhibitory effect of the processing of target-like distracters has already been shown to affect the conscious detection of simple motion and simple orientation stimuli in a random dot kinematogram. In two experiments we examined the effects of single-feature motion distracters, single-feature orientation distracters, and combined-feature distracters containing both motion and orientation information. The target was specified as a coherent motion episode (Experiment 1) or as a combined-feature episode where the coherent motion was accompanied by an abrupt change in line orientation (Experiment 2). Results showed that (a) the respective feature-specific inhibitory processes operate separately even when the distracter features are presented simultaneously and (b) both inhibitory processes contribute to the blindness effect when the conjunction of two features is defined as the target. Again, this inhibitory-process is feature-specific: Only features that are defined in the task are represented in the inhibitory task set. In case of combined- feature task-sets, these representations remain separate, so that combined-feature distracters as well as single-feature distracters are able to induce blindness effects.

## INTRODUCTION

The conscious perception of basic visual features depends on the attentional resources available to the system (Rees, Frith, & Lavie, 2001). In a series of experiments, we previously demonstrated that access and processing of relevant information is not only affected by the presentation of rivaling information, but also by distracters preceding the target stimulus (Michael, Hesselmann, Kiefer, & Niedeggen, 2011; Sahraie, Milders, & Niedeggen, 2001). In our paradigm, two spatially separate rapid serial visual presentation (RSVP) sequences are shown. In a local stream, the color of a central fixation point changes at 10 Hz. The surrounding area consists of a random dot kinematogram (RDK) whose dots follow a random walk. The random global motion is interrupted by salient events like short episodes of coherent motion for 100 ms. The subject’s task is to detect the onset of a coherent motion coinciding or following a red fixation. Thus, the color change in the local stream serves as a cue to shift attention to the global stream. Task-irrelevant motion episodes or orientation changes presented prior to the cue serve as distracters and have to be ignored.

In the original attention-induced motion blindness paradigm (Sahraie et al., 2001), the detection of coherent motion episodes (target) in an RDK was severely impaired when coherent motion episodes were presented prior to the target epoch (as distracters). Moreover, the detection rate for the targets critically depends on the frequency of the distracters (Hesselmann, Niedeggen, Sahraie, & Milders, 2006; Sahraie et al., 2001). In a variation of this paradigm, a similar effect could be obtained when the dots in the RDK were replaced by short lines of the same orientation. Here, abrupt orientation changes of the lines defined target and distracters. As obtained in our previous experiments, the presence of distracters affected conscious access to the target. Since detection rate critically depends on the number of distracter episodes preceding the target, we assumed that a similar mechanism is involved (Michael et al., 2011). We will refer to this effect as *distracter induced blindness* (DIB).

The effect appears to be resolved within 200-300 ms following the onset of the cue. An increase of detection rate for the target with increasing cue-target stimulus onset asynchrony (SOA) can be obtained with a ceiling effect at about 300 ms (Sahraie et al., 2001). We hypothesized that distracters activate a central suppression mechanism which prevents that visual features - not relevant at the time of presentation - will be updated. The occurrence of the cue triggers the release of this inhibition which appears to be an inertial process so that distracter-induced blindness is fully released at approximately 300 ms (Hesselmann et al., 2006; Hesselmann, Allan, Sahraie, Milders, & Niedeggen, 2009; Sahraie et al., 2001).

The characteristics of DIB resemble the properties of the attentional blink. In both paradigms, the detection of a second target critically depends on its temporal distance to a primary target (Shapiro, Raymond, & Arnell, 1994). As in DIB, the performance in the attentional blink is also modified by distracter-like events (Maki & Padmanabhan, 1994; Zhang, Zhou, & Martens, 2009). An explanation for the distracter effects in the attentional blink provided by Zhang et al. (2009) resembles the suppression model by Sahraie et al. (2001). Zhang et al. assumed a negative attentional set as suggested by our previous studies (Niedeggen, Sahraie, Hesselmann, Milders, & Blakemore, 2002; Sahraie et al., 2001), which is triggered by distracters perceptually and/or semantically similar to the target. Zhang et al. (2009) claimed that the negative attentional set is defined at an abstract categorical level and it is thus category-specific. In three experiments, Zhang et al. showed that the detection of an Arabic digit target in an RSVP sequence of black letters is impaired when additional distracters share the semantic category (Arabic digits or Chinese number characters). The detection of the target was not affected, when symbols were presented as perceptually and categorically deviant distracters.

The activation of such negative attentional sets (or the inhibition of task sets) is well known to modulate the processing of specific stimulus features (Kiefer & Martens, 2010; Kiesel, Kunde, & Hoffmann, 2007; Mayr, Diedrichsen, Ivry, & Keele, 2006; Mayr & Keele, 2000). Task sets are defined as top-down processes that control attentional target selection when target-defining features are specified in advance. Their function is to accelerate target processing, resolve target competition, and inhibit the processing of irrelevant stimuli in working memory (Eimer, Kiss, & Nicholas, in press). It is therefore possible that irrelevant motion distracters, which have to be ignored, will inhibit a motion task-set, and orientation distracters a corresponding orientation task-set. This is in line with our previous experiments, which demonstrated that the inhibition process is feature-specific (Michael et al., 2011). We also observed that both task sets can be inhibited independently if the target is defined by two visual features (either motion onset or orientation switch). The independence of attentional and task sets, respectively, has already been demonstrated in other experiments (e.g., Mayr & Keele, 2000).

**Figure 1.**
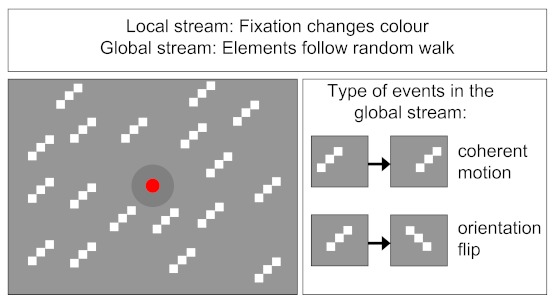
Schematic diagram showing the properties of the local and global RSVP (rapid serial visual presentation) stream.

In the current study, we examined whether the feature-specific inhibition obtained in our previous studies only depends on the a priori task set, or whether the visual features can also be combined in one “distracter episode”. In our previous experiment, the distracter episode was always defined by the presentation of a single feature (motion or orientation) in the pre-cue epoch (see Figure 1). In Experiment 1, we now included the conjunction of two different visual features in a distracter episode (motion and orientation), while the target was still defined by a single feature (task set: motion). There is evidence that task-set inhibition only occurs in the context of endogenous activation of a new task, whereas no inhibition takes place when the new task is unpredictable or the stimulus is irrelevant (Hübner, Dreisbach, Haider, & Kluwe, 2003). Moreover, the presence of two features rather than one feature as distracters showed that there is no additional blindness effect (Michael et al., 2011).

According to these findings, we expect that only the motion feature will lead to a DIB effect: As the target is defined by motion, preceding orientation changes are always irrelevant. In case of conjoined features, the additional presence of orientation changes increases the comple-xity of the distracter episode, but does not lead to changes in DIB. If the task set is inhibited specifically by motion, the degree of inhibition should not be affected by the additional presence of orientation flips.

## Experiment 1

In this experiment, we tested the notion of feature-specific negative attentional sets and used a simultaneous presentation of coherent motion and orientation changes as a combined-feature distracter condition in addition to pure motion and pure orientation distracters (single-feature distracters). If the negative attentional set that produces the DIB effect is feature-specific and activated endogenously, the detection of motion targets is expected to be impaired, when distracters contain motion information whether presented as single-feature motion distracters or as combined-feature distracters (Hübner et al., 2003; Michael et al., 2011).

### Participants

The sample consisted of 11 participants (9 females and 2 males) with an age-range of 21 to 34 years (M_age_ = 25.82, *SD* = 4.38) and with normal or corrected-to-normal vision. All subjects were students of the Freie Universität Berlin and were recruited by advertisement. They did not receive payment but were given course credit after giving informed consent. Two additional individuals participated but were excluded from further analysis due to high error rates in the no target condition (false alarms > 80%).

### Procedure

The subjects sat in a comfortable chair with the head 57 cm in front of the computer monitor, within a constantly lit, sound-reduced, and air-conditioned cubicle. The stimuli were presented on a 21-inch CRT monitor with a screen resolution of 2,048 × 1,536 pixels at 100 Hz. The Visual Stimulus Generator (VSG; Cambridge Research Systems Ltd., Kent, UK) was used to generate and control the stimuli.

The local stream consisted of a central fixation point (0.5° dia-meter), changing its colour (blue, green, yellow each bright and dark and three different luminance grey levels) randomly every 100 ms, surrounded by a grey circular patch (3.5° diameter). In the global stream, 150 white lines moved randomly on a dark grey background (25° × 25°). Each line consisted of three squares (0.18° diameter) that were arranged diagonally (for a schematic view, see Figure 1). All lines were always oriented equally.

In each trial, subjects attended to the colour red in the local stream, which served as the cue for the target task to detect the presence and direction of a coherent motion in the global stream. Episodes of coherent motion and/or abrupt changes in orientation (diagonally left-to-right to diagonally right-to-left and vice versa) were presented as distracter events prior to the cue (single-feature vs. combined-feature distracters) and had to be ignored. The target appeared between 1,500 and 2,500 ms after the beginning of each trial. The trial length was always 3,500 ms. In order to ease the temporal separation of distrac-ters and targets, distracters were not presented in an interval of 400 ms prior to the cue, whereas at least one distracter appeared 400 to 700 ms before the cue in order to induce temporal uncertainty. The remaining distracters were presented at a randomly determined time point between 400 ms after the beginning of the trial and 400 ms prior to the cue. The directions of the coherent motion episodes were always horizontal. Directly succeeding motion episodes were characterized by opposite motion directions in order to maintain a motion onset. After each trial, a signal tone summons the subject to report whether a target was detected or not by button presses on a response box. In addition to the presence of a target, the direction of the detected motions should be indicated. Direction discrimination was only assessed when the target was detected. Only those trials entered the analysis as detected successfully in which coherent motion was detected and its direction was correctly discriminated.

For each participant, one block of trials was presented. All of the 240 trials were presented in randomized order. In each 90 trials, the SOA between the onset of the cue and the target was 0 ms or 300 ms, respectively. For each SOA, three different distracter conditions were defined including each 30 trials: (a) six coherent motion episodes were presented prior to the cue (single feature: task relevant), (b) six changes in orientation were presented (single feature: task irrelevant), and (c) six combined episodes of motion coherence and orientation change (combined feature: relevant and irrelevant). Two additional control conditions were included (30 trials without target presentation and 30 trials without presentation of the cue) in order to control the response bias of the participants. The whole experiment lasted approximately 30 min.

### Results

Trials without cues were detected successfully by all participants (mean rate 99.7%); trials without targets led to a false alarm rate of 11.8% (*SD* = 8.9%, range 0-30%).

**Figure 2.**
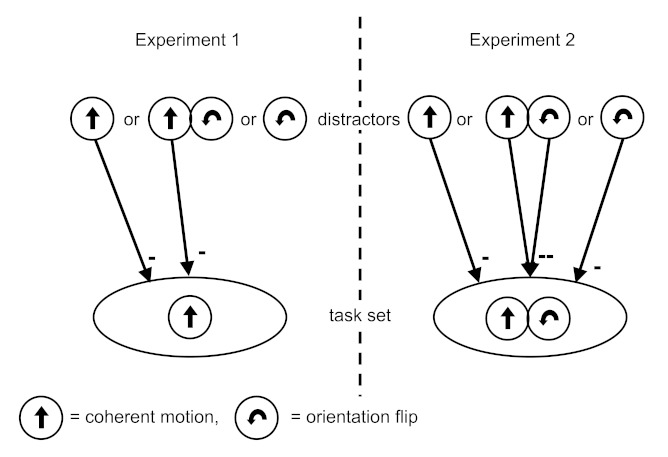
Results of Experiments 1 and 2: Target detection rates for trials with single-feature motion distracters (grey bars), single-feature orientation distracters (white bars) and combined-feature distracters (hatched bars) presented prior to the cue. Error bars indicate standard errors of the means. SOA = the stimulus-onset asynchrony.

Analysis of the experimental conditions SOA and Distracters (see Table 1) indicates an effect of SOA as well as a feature-specific effect: Detection rate was low when the target was presented simultaneously with the cue, and when single-feature motion distracters or combined-feature distracters were presented (see Figure 2).

**Table 1.** Means of Motion Detection Rates.

Distracters	SOA 0 ms	SOA 300 ms
Motion	45.15 (7.62)	71.52 (6.57)
Orientation	68.18 (10.91)	82.73 (9.07)
Combined	40.00 (8.28)	72.42 (7.98)

*Note*. Standard error of the mean in parentheses. SOA = the stimulus-onset asynchrony.

A 2 × 3 ANOVA for repeated measures for the factors SOA (0 vs. 300 ms) and Distracters (single-feature: motion vs. single-feature: orientation vs. combined-features: motion and orientation) confirmed this impression: In all distracter conditions, detection rate increased significantly with increasing cue-target SOA, *F*(1, 10) = 59.02, *p* < .001, η^2^_p_ = .86. Mere presentation of task-irrelevant orientation distracters enhanced motion detection in comparison to distracter conditions including motion episodes (single-feature motion and combined-feature distracters), and expressed itself in a main effect of Distracters, *F*(2, 20) = 9.14, *p* =.007, η^2^_p_ = .65. The interaction SOA × Distracters, *F*(2, 20) = 5.83, *p* =.013, η^2^_p_ = .37, indicated that the aforementioned effect was more pronounced at SOA of 0 ms (see Figure 2): Here, target detection was significantly lower in trials with single-feature motion distracters, *t*(10)=2.83, *p* =.02, as well as in trials with combined-feature distracters, *t*(10) = 3.66, *p* < .01, as compared to trials with single-feature orientation distracters. At an SOA of 300 ms, these differences were less pronounced; compare single-feature motion vs. single-feature orientation, *t*(10) = 2.17, *p* =.06; and combined-features vs. single-feature orientation, *t*(10) = 3.00, *p* =.01. In no SOA condition, differences between single-feature motion and combined-features distracters gained significance.

These findings clearly indicate that experimentally induced blindness is significantly modulated by distracters sharing the target’s feature. Motion detection was severely impaired by both single-feature motion distracters and combined-feature motion and orientation distracters. In contrast, motion detection was least impaired when mere orientation changes were presented as distracters. The results show that the distracters evoke a feature-specific inhibitory attentional set that impairs perception of targets, which share this very feature. The fact that combined-feature distracters, although they are perceptually dissimilar to the target, induce a comparable inhibitory attentional set as single-feature motion distracters suggests that task set inhibition occurs in the context of endogenous control. This process is not disturbed by additional inhibitory processes because the additional visual feature in this experiment is always irrelevant for the task and therefore causes no interference and does not lead to an additional inhibition (Hübner et al., 2003).

## Experiment 2

Experiment 1 provided evidence that combined features in the distracter episode have no different effects on target processing than single-feature distracters. In other words, the system responds only to the visual features which are defined a priori in the task set. Therefore, we changed the number of visual features critical for the task set in our second experiment: Here, the simultaneous presentation of both features (coherent motion and change in orientation) was defined as the target whereas the mere presentation of a single feature was to be ignored. As in Experiment 1, distracter episodes were defined by single-feature events (motion, orientation) and by combined feature events (motion and orientation).

In contrast to Experiment 1, the distracter episodes always shared at least one of the targets’ features. As Experiment 1 showed, the DIB effect is feature-specific, even when the combined-feature distracters are perceptually dissimilar to the motion target. Therefore, we assume that the strength of task-set inhibition depends on the match of the distracter and the target episode: When the conjunction of both features is defined as targets, both types of single-feature distracters match to a part of the target’s features. This should lead to a partial inhibition of the task set. The conjunction of features, however, is known to produce stronger effects compared with single features (Lavie, 1997). Therefore, combined-feature distracters should lead to a maximal DIB effect.

### Participants

The sample consisted of 12 new participants (5 females and 7 males) with an age-range of 21 to 34 years (M_age_ = 25.67, *SD* = 4.56) and with normal or corrected-to-normal vision. All subjects were students of the Freie Universität Berlin and were recruited by advertisement. They did not receive payment but were given course credit after giving informed consent. Two additional individuals participated but were excluded from further analysis due to high rates of motion detection in the no target condition (false alarms > 80%).

### Procedure

In Experiment 2, the same temporal arrangement of targets and distracters as in Experiment 1 was used again. For each participant, one block of trials was presented. All of the 520 trials were presented in randomized order. Targets were defined as coherent motion episodes accompanied by a switch of line orientation, whereas the mere presentation of motion episodes or orientation changes simultaneous to or after the cue was defined as irrelevant and had to be ignored. In each of the 120 trials, the SOA between the onset of the cue and the target was 0 ms or 300 ms, respectively. For each SOA, three different distracter conditions were defined including each 40 trials: (a) six coherent motion episodes were presented prior to the cue, (b) six changes in orientation were presented, and (c) six combined episodes of motion coherence and orientation change. The mere presentation of motion episodes or orientation changes simultaneous to or after the cue occurred each in another 60 trials with a cue-target SOA of 0 ms and another 60 trials with a cue-target SOA of 300 ms. Again, each distracter category was presented in one third of trials. As an additional control condition, 40 trials without cue were presented. The whole experiment lasted approximately 60 min.

### Results

Trials of the no-cue condition were presented to ensure the fixation of the local stream, these events were detected successfully (mean rate 97.1%) by all participants. Trials without targets led to a false alarm rate of 16.5% (*SD* = 10.8 %, range 1-44%). The results for the experimental conditions SOA and Distracters (see Table 2 and Figure 2) showed lowest detection rates for the short SOA, and if combined distracters were presented.

**Table 2.** Means of Motion Detection Rates.

Distracters	SOA 0 ms	SOA 300 ms
Combined	30.83 (6.92)	52.71 (7.07)
Motion	36.46 (7.50)	57.50 (8.66)
Orientation	40.21 (7.65)	62.71 (6.64)

*Note*. Standard error of the mean in parentheses. SOA = the stimulus-onset asynchrony.

A 2 × 3 ANOVA for repeated measures for the factors SOA (0 ms vs. 300 ms) and Distracters (motion vs. orientation vs. motion and orientation) confirmed this impression: In all distracter conditions, SOA of 300 ms led to higher detection rates than SOA of 0 ms, *F*(1, 11) = 41.85, *p* < .001, η^2^_p_ = .79. Combined-feature distracters led to lowest detection rates compared to single-feature distracters, resulting in a main effect Distracters, *F*(2, 22) = 6.03, *p* =.016, η^2^_p_ = .35, corrected by Greenhouse-Geisser, = .759. Bonferroni-corrected pairwise comparisons showed that this effect arises due to the differences between combined-feature distracters and single-feature motion distracters (*p* =.049) and between combined-feature distracters and single-feature orientation distracters (*p* =.031). The differences between single-feature motion and single-feature orientation distracters did not gain any significance. A significant interaction SOA × Distracters was also not obtained.

In this experiment, the target was defined by a combination of motion coherence and orientation change. Assuming the aforementioned inhibition model, the inhibition of the task set is maximal when combined-feature distracters are presented prior to the cue and a perfect match between distracter and target features is given. In case of single-feature distracters, only the inhibition of one feature is triggered and the task set is inhibited less due to the only partial match between distracter and target features.

Also the rate of false alarms for task-irrelevant features at the target position supports this view. Following the idea that the presentation of single-feature distracters triggers an inhibitory process that causes DIB when the same feature is presented as the target, participants tend to specifically be blind for this feature. In trials where only a single feature is presented simultaneously to or after the cue and the same feature has been presented as a distracter, participants have the impression of either having perceived only a single feature or of having seen nothing. Boththese effects are due to DIB which makes it easy for the participants to classify the trial as “no target”. In case of a distracter induced inhibition which does not match the feature at the target position, target processing should be undisturbed, leading to a stronger visual impression of the respective uninhibited feature. Possibly a higher uncertainty concerning the additional presence of another, distracter-inhibited feature led to higher rates of false alarms in these trials.

Indeed, the data confirmed that it is harder for the participants to reject a single feature, when the respective other feature was presented as a distracter (see Table 3). A 2 × 2 ANOVA for repeated measures with the factors Feature at Target Position (motion vs. orientation) and Distracters (motion vs. orientation) shows an interaction of Feature at Target Position × Distracters, *F*(1, 11) = 29.25, p < .001, η^2^_p_ = .73.

**Table 3.** Means of False Alarms of Irrelevant Events at the Target Position.

	Feature at target position	
Distracters	Motion	Orientation
Motion	12.29 (3.66)	21.04 (4.60)
Orientation	27.08 (5.48)	5.63 (1.63)

*Note*. Standard error of the mean in parentheses.

## General Discussion

The inhibitory effect of distracter processing has already been shown to affect the conscious detection of simple motion stimuli (AMB, attention-induced motion blindness; Sahraie et al., 2001). Our current findings extend these results by showing that (a) the respective feature-specific inhibitory processes operate separately even when the distracter features are presented simultaneously and (b) both inhibitory processes contribute to the blindness effect when the conjunction of two features is defined as target. Again, this inhibitory process is feature-specific.

In our first experiment, the effect of feature-specific inhibition on the processing of motion targets was examined; detection of motion targets was impaired as soon as the distracters contained motion information. Following the suppression model by Sahraie et al. (2001), two separate inhibitory mechanisms were established by the visual features which served as distracter events in this experiment. We showed that blindness effects for motion stimuli arise by a suppression of processing motion events due to an inhibition of irrelevant motion distracters. In case of distracters consisting of mere orientation changes, the processing of orientation changes will be gradually more suppressed the more distracters are presented. Given that orientation changes never served as a target event in this experiment, this specific inhibition never came to an effect.

**Figure 3.**
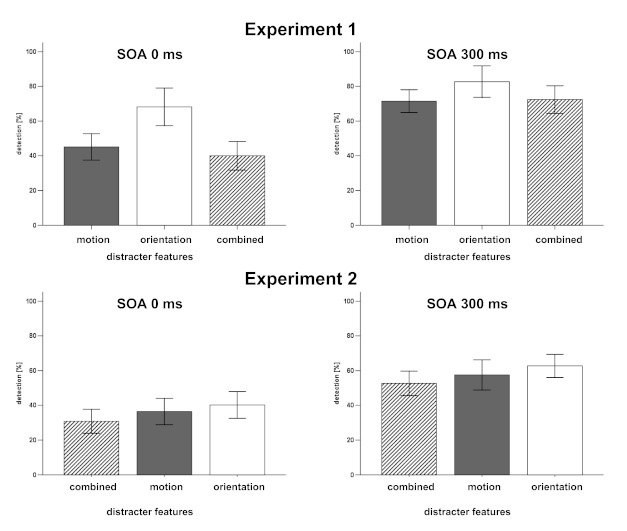
A model of task set inhibition due to distracters. In Experiment 1, motion distracters and combined distracters are able to inhibit the motion task-set. In Experiment 2, motion and orientation distracters only match partially with the combined task-set, inhibition is at a maximum for combined distracters.

In Experiment 2, the task set consisted of both motion coherence and orientation change information. The inhibition of this task set was maximal when combined-feature distracters were presented prior to the cue and a perfect match between distracter and target features was given. In case of single-feature distracters, only the inhibition of one feature was triggered and the task set was inhibited less due to the only partial match between distracter and target features. The findings lead to a model depicted in Figure 3. Following the ideas of Hübner et al. (2003), task-set inhibition only occurs due to endogenous controlled processes to avoid interference. This avoidance was not necessary in Experiment 1, since orientation information was not defining the target event. Also the combination of (inhibited) motion information with orientation flips showed no additional effects which rules out the possibility of an exogenous, distracter-driven generation of inhibition: Only distracter information that is specified in the task-set led to feature-specific inhibitory processes. Motion distracters and combined distracters were similarly able to inhibit the motion task-set.

The results of Experiment 2 again suggested that task-set inhibition occurred in the context of endogenous control. The processes seemed to be independent (even at task-set level) since the different single-feature distracters led to comparable effects: Motion and orientation distrac-ters only partially matched with the combined task set, and inhibition was maximal for combined distracters.

These results are well in line with recent findings. For example, in a study by Tapia, Breitmeyer, and Shooner (2010), participants were instructed to attend and respond to form, color, or the combination of form and color of a mask probe that followed either an invisible (masked) or a visible (unmasked) prime. Results indicate that increased reaction times are only due to incongruent and task-relevant primes whereas irrelevant primes do not contribute to this effect, even when they are incongruent. Comparable to our results, the task set is inhibited specifically by the feature that defines the target. In the se-cond experiment, Tapia et al. (2010) ruled out that effects in a conjoint task (where both color and form of a stimulus were attended) could be attributed to the mere presence of two features. In fact, the conjunction of both features is important for the effects. In this experiment, the probe was flanked by two primes. In a conjoint feature condition, color and form were conjoined in one of the two flanking stimuli. In a disjoint condition, color and form were presented separately, each in one of the two flanking stimuli. The results showed clearly that the conjunction is crucial for stronger priming effects. Although this is only true in the visible condition, these mechanisms seem to be comparable to our model: In our experiments, distracter and target episodes were also unmasked and visible. In contrast to Tapia et al., however, the detection of our targets was not slowed, but their visibility decreased.

In an earlier investigation (Michael et al., 2011), we found the detection of orientation targets was almost flawless without preceding distracters. This finding is comparable to the detection of motion targets without preceding distracters reported by Sahraie et al. (2001).

In the present study, we neglected this control condition since we were primarily interested in the comparison of two different distracter features. Nevertheless, the control would have been helpful for the evaluation of the data of Experiment 2. The discrimination of the combined-feature target is assumed to reduce the detection performance - even if no distracters are presented. This assumption is supported by the lower overall detection performance in Experiment 2 compared with Experiment 1.

Furthermore, it remains unclear whether the irrelevant orientation distracters in Experiment 1 led to an inhibitory process or not: Changes in orientation did not contribute to the distracter-induced motion blindness effect. Here, two mechanisms are plausible. The first one assumes that the irrelevant orientation distracters lead to an inhibition of orientation processing. As no orientation targets were presented, and therefore orientation is not part of the task set, this inhibition had no effect. In earlier experiments (Michael et al., 2011), we showed that such an inhibition takes effect when orientation targets occur, even if they are unpredictable. However, in these experiments the target was defined as being either a motion episode or a change in line orientation. Therefore, orientation was also part of a more complex task set. Alternatively, one can also consider the second explanation, that as long as the task-set is defined only by motion, all other events do not lead to inhibition because they are not inhibited by a top-down controlled attentional set. In this case, it is possible that any kind of visually salient event captures attention bottom-up, regardless of whether it is task-relevant or not. The task set then controls the disengagement from these irrelevant features (e.g., Theeuwes, 2010). We can rule out the latter possibility by the feature specificity of DIB: Salient events only lead to task-set inhibition when they are specified in the task. Similar findings were also reported by other authors (e.g., Folk, Remington, & Johnston, 1992).

In sum, our results indicate that the DIB effect is due to feature-specific inhibitory processes that operate separately even when the distracter features are presented simultaneously. When the conjunction of two features is defined as a target, both inhibitory processes contribute to the blindness effect. Again, this inhibitory process is feature-specific: Experiment 1 showed that only the target-defining feature is represented in the task set. The results of Experiment 2 suggest that the representation of both single-feature events implied by the task set are maintained separately and can be inhibited by both single-feature and combined-feature distracters.
